# Enrichment of ^13^C in diacids and related compounds during photochemical processing of aqueous aerosols: New proxy for organic aerosols aging

**DOI:** 10.1038/srep36467

**Published:** 2016-11-04

**Authors:** Chandra Mouli Pavuluri, Kimitaka Kawamura

**Affiliations:** 1 Institute of Surface-Earth System Science, Tianjin University, Tianjin 300072, China; 2 Institute of Low Temperature Science, Hokkaido University, Sapporo 060-0819, Japan

## Abstract

To investigate the applicability of compound specific stable carbon isotope ratios (δ^13^C) of organics in assessment of their photochemical aging in the atmosphere, batch UV irradiation experiments were conducted on two ambient (anthropogenic and biogenic) aerosol samples in aqueous phase for 0.5–120 h. The irradiated samples were analyzed for δ^13^C of diacids, glyoxylic acid (ωC_2_) and glyoxal. δ^13^C of diacids and related compounds became larger with irradiation time (i.e., aging), except for few cases. In general, δ^13^C of C_2_-C_4_ diacids showed an increasing trend with decreasing chain length. Based on δ^13^C of diacids and related compounds and their relations to their concentrations, we found that C_2_ and C_3_ are enriched with ^13^C during the photochemical decomposition and production from their higher homologues and oxoacids. Photochemical breakdown of higher (≥C_3_) to lower diacids is also important in the enrichment of ^13^C in C_3_-C_9_ diacids whereas their production from primary precursors causes depletion of ^13^C. In case of ωC_2_ and glyoxal, their photochemical production and further oxidation to highly oxygenated compounds both cause the enrichment of ^13^C. This study reveals that δ^13^C of diacids and related compounds can be used as a proxy to trace the aging of organic aerosols during long-range atmospheric transport.

Organic aerosols (OA), which represent a large fraction of fine particles^1^, play an important role in atmospheric processes and have serious impacts on the Earth’s climate system and human health^2^,^3^,^4^,^5^. OA are emitted directly into the atmosphere from various primary sources and secondarily produced in the atmosphere by oxidation of volatile organic compounds (VOCs) followed by condensation on pre-existing particles and/or nucleation^1^,^6^. The oxidation products are highly water-soluble and typically contain several types of functional groups including carboxylic acids, carbonyls, and hydroxyl groups as well as peroxides^7^,^8^. In the presence of moisture (i.e., clouds and fog), water-soluble compounds can partition into the aqueous phase and react with dissolved oxidants or undergo direct photolysis by actinic radiation^9^,^10^, thus OA are subjected to photochemical processing (aging) for several days until they are removed from the atmosphere through wet and/or dry deposition^11^,^12^,^13^. It is well known that aqueous phase oxidation processes can be very different from those in gas phase. Most of the recent studies have been focused on the ability of aqueous phase reactivity of selected organic compounds and other studies on the effects of aging of complex organic mixtures in aqueous aerosols^9^,^10^,^11^,^12^,^13^,^14^,^15^,^16^,^17^,^18^,^19^. However, identification of proxies, except for some molecular tracers^9^, for the assessment of aging of OA during long-range atmospheric transport remains unclear yet.

Low molecular weight dicarboxylic acids and related polar compounds are known as ubiquitous in atmospheric aerosols and atmospheric waters^20^,^21^,^22^,^23^,^24^. Although diacids, oxoacids and α−dicarbonyls can be emitted directly into the atmosphere from primary sources such as incomplete combustion of fossil fuels^25^ and biomass burning^26^, they are mainly produced by secondary processes of VOCs of both anthropogenic and biogenic origin^14^,^16^,^19^,^27^,^28^,^29^. They are further subjected to significant photochemical oxidation in the atmosphere during long-range transport; e.g., carbonyls to carboxylic acids^30^ and breakdown of higher to lower diacids^16^,^31^. The molecular distributions of diacids have been considered to understand aging of organic aerosols in the atmosphere^21^.

Stable carbon isotopic composition (δ^13^C) of specific organic compound can provide highly valuable information on its aging in the atmosphere^32^,^33^,^34^,^35^, because the isotopic fractionation of carbon occurs upon chemical reactions or phase transfer^35^. Unidirectional reactions show a preferential enrichment of ^12^C in reaction products with the remaining reactants being isotopically heavier^36^. On the other hand, chemical processing within the particle (or aqueous) phase causes the enrichment of ^13^C in the reactant retained in the same phase, if some of the reaction products are volatile that are isotopically lighter^37^. It was found that remaining aliphatic/aromatic hydrocarbons including isoprene become more enriched with ^13^C after photochemical oxidation with hydroxyl radical (HO^•^) in laboratory experiments and field measurements^32^,^33^. It was also found that ^13^C is enriched in the remaining oxalic acid (C_2_) during the photolysis catalyzed by Fe^3+^ (and Fe^2+^) in aqueous phase in a laboratory experiment^34^. Therefore, it is very likely that δ^13^C of diacids and related compounds could be used as a proxy for assessing the aging of organic aerosols in the atmosphere.

Wang and Kawamura^38^ found a decrease in the concentrations and an increase in δ^13^C of saturated diacids, especially C_2_-C_4_ diacids, in marine aerosols from midlatitudes toward the equator over the western Pacific. In addition, they reported an increasing trend in the relative abundance of C_2_ in total diacids (C_2_%), a measure of aging^21^ and interpreted the enrichment of ^13^C in diacids toward the equator due to the kinetic isotope effects (KIE) caused by photochemical degradation. Pavuluri *et al*.^39^ also reported higher δ^13^C of C_2_-C_4_ diacids in the tropical Indian aerosols with an increase in δ^13^C from C_4_ to C_2_, which are considered to be aged during long-range transport from distant source regions. They interpreted an enrichment of ^13^C in C_2_-C_4_ diacids as a result of photochemical breakdown of higher to lower diacids and C_2_ diacid to CO_2_ during long-range atmospheric transport, despite a linear relationship between concentrations of C_2_ and C_2_%. However, there are no laboratory studies on the changes in δ^13^C of organic compounds with aging under atmospherically relevant conditions.

In this study, we conducted batch UV irradiation experiments using two types of ambient aerosol samples, which represent anthropogenic (AA) and biogenic aerosols (BA), collected from Chennai, India in the presence of moisture for different time periods from 0.5 to 120 h to better understand the applicability of δ^13^C of diacids, oxoacids and α-dicarbonyls in assessing the atmospheric aging of OA. Here, we report the changes in δ^13^C of diacids, glyoxylic acid and glyoxal as a function of irradiation time. Based on the observed results together with the changes in their concentrations, we discuss possible photochemical processes responsible for an enrichment or depletion of ^13^C in diacids and related compounds with the aging of aqueous aerosols, thus infer the use of δ^13^C of diacids and related compounds as a proxy for photochemical aging.

## Results

Table 1 presents δ^13^C of normal saturated α,ω-diacids (C_2_-C_6_ and C_9_) and aromatic diacid (phthalic acid, Ph), glyoxylic acid (ωC_2_) and glyoxal (Gly) determined in non-irradiated AA and BA samples as well as their average and median values in the irradiated (0.5 to 120 h) AA and BA samples. On average, δ^13^C of C_2_, C_3_, Ph, ωC_2_ and Gly in irradiated AA and BA are higher than those of non-irradiated AA and BA samples (Table 1). Further, δ^13^C of C_2_ is highest followed by C_3_ and C_4_ in both non-irradiated and irradiated (on average) AA and BA (Table 1). In contrast, average δ^13^C of C_4_ and C_5_ diacids in irradiated AA and BA samples are lower than those of non-irradiated samples. C_6_ diacid shows an enrichment of ^13^C in irradiated samples whereas C_9_ presents a depletion of ^13^C in irradiated samples, compared to that in non-irradiated samples (Table 1).

**Figure 1:**
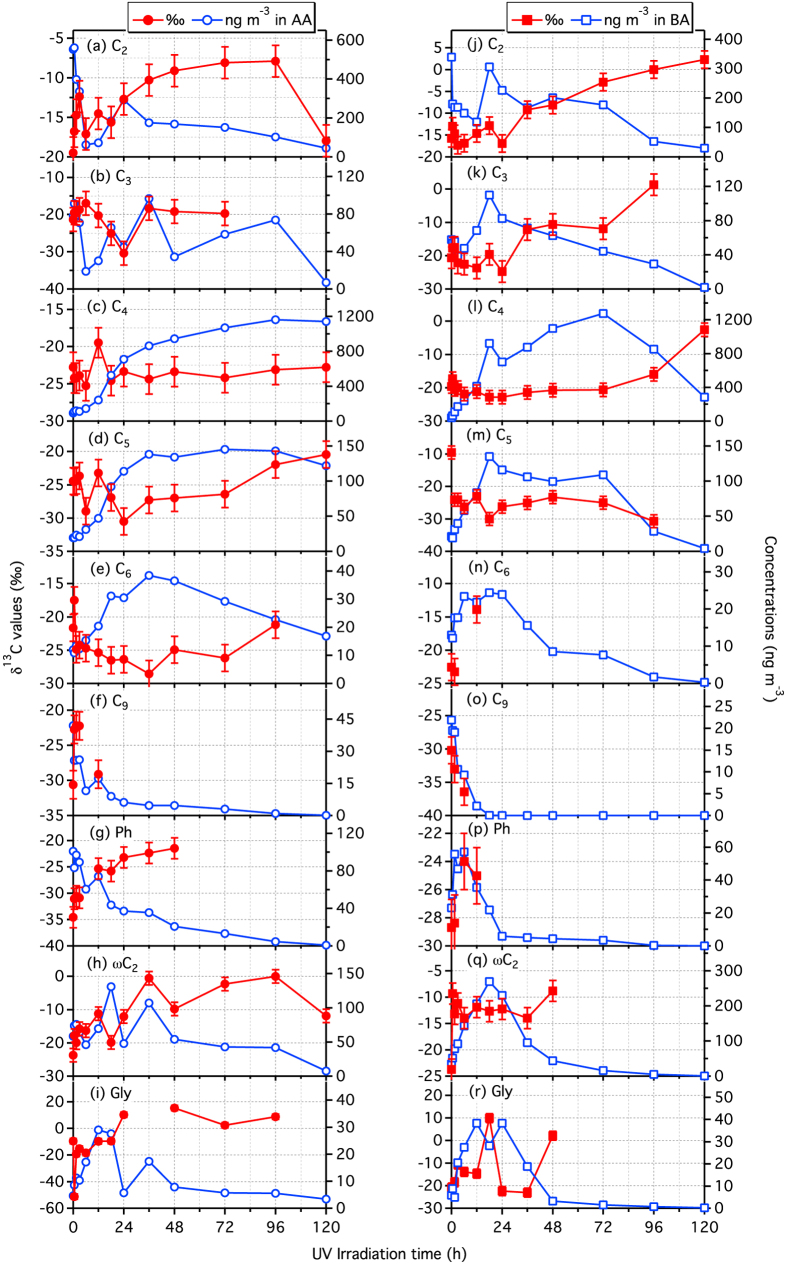
Changes in δ^13^C of diacids (C_2_-C_6_, C_9_ and Ph), glyoxylic (ωC_2_) acid and glyoxal (Gly) together with the changes in their concentrations with UV irradiation time in AA and BA. Concentrations data is obtained from Pavuluri *et al*.^19^. Bars show the experimental errors in δ^13^C of diacids and related compounds.

Figure 1 shows changes in δ^13^C of diacids and related compounds together with changes in their concentrations as a function of UV irradiation time. We found that δ^13^C of C_2_ diacid increase with irradiation time, except for few points, in both AA and BA, although its concentrations declined (Fig. 1a,j). δ^13^C of C_3_ diacid also increased, being opposite to its concentrations in both samples, although the trend is not clear during early stages of irradiation in the case of BA (Fig. 1b,k). Interestingly, δ^13^C of C_4_ showed a general decrease with irradiation up to 6 h followed by an increase until the end of in both AA and BA experiments, except for few cases, despite a significant increase in its concentrations with irradiation up to 72 h in BA and 96 h in AA (Fig. 1c,l). However, the enrichment of ^13^C in C_4_ diacid was small in both samples, except for later stages of irradiation (72 to 120 h) in BA when the concentration of C_4_ decreased drastically (Fig. 1c,l).

Similarly, δ^13^C of C_5_ diacid decreased with irradiation time up to 18 h in BA and 24 h in AA, except for few cases, and then increased until the end of AA experiment and up to 48 h in BA experiment followed by a decrease thereafter (Fig. 1d,m). δ^13^C of C_6_ diacid in AA also decreased with time up to 36 h and then increased, except for few points, until the end of experiment (Fig. 1e). In case of BA, C_6_ was depleted with ^13^C in 1.5 h and enriched with ^13^C in 12 h compared to non-irradiated sample (Fig. 1n). Such trends of δ^13^C of C_5_ and C_6_ are opposite to those of their concentrations, except for few points (Fig. 1d,e,m,n). Being consistence with other normal diacids, C_9_ diacid in AA showed an enrichment of ^13^C with irradiation, except for 12 h (Fig. 1f). On the contrary, δ^13^C of C_9_ in BA showed a decrease with irradiation, despite a decrease in its concentration (Fig. 1o). δ^13^C of Ph also showed an increase with time in both AA and BA samples (Fig. 1g,p). δ^13^C of ωC_2_ and Gly in AA showed a general increase with time, although their concentrations increased during early stages of irradiation (i.e., up to 24 h) and decreased gradually during later stages of irradiation (Fig. 1h,i). They did not show clear trends in the case of BA (Fig. 1q,r).

## Discussion

We generally found an inverse relation between δ^13^C and concentrations of individual diacids and related compounds, although correlations are weak in most cases (Fig. 1, Table 2). This trend is consistent with that reported in remote marine aerosols from midlatitudes toward the equator over the western Pacific^38^. The decrease in C_2_ and C_3_ diacid concentrations with irradiation time (i.e., aging) has been attributed to the overwhelming photolysis catalyzed by water-soluble iron^19^, because C_2_ and C_3_ diacids tend to form a complex with Fe^3+^ (and C_2_ even with Fe^2+^) by acting as ligands in aqueous phase and then both the diacids photolyze upon the absorption of UV light to result in Fe^2+^ and CO_2_^40^,^41^,^42^,^43^. Fe^2+^-oxalato complex undergoes a charge transfer from the Fe^2+^ to the surrounding solvent molecule, upon the absorption of UV light, forming Fe^3+^-oxalato complex, and then results in Fe^2+^ and CO_2_^41^.

**Figure 2:**
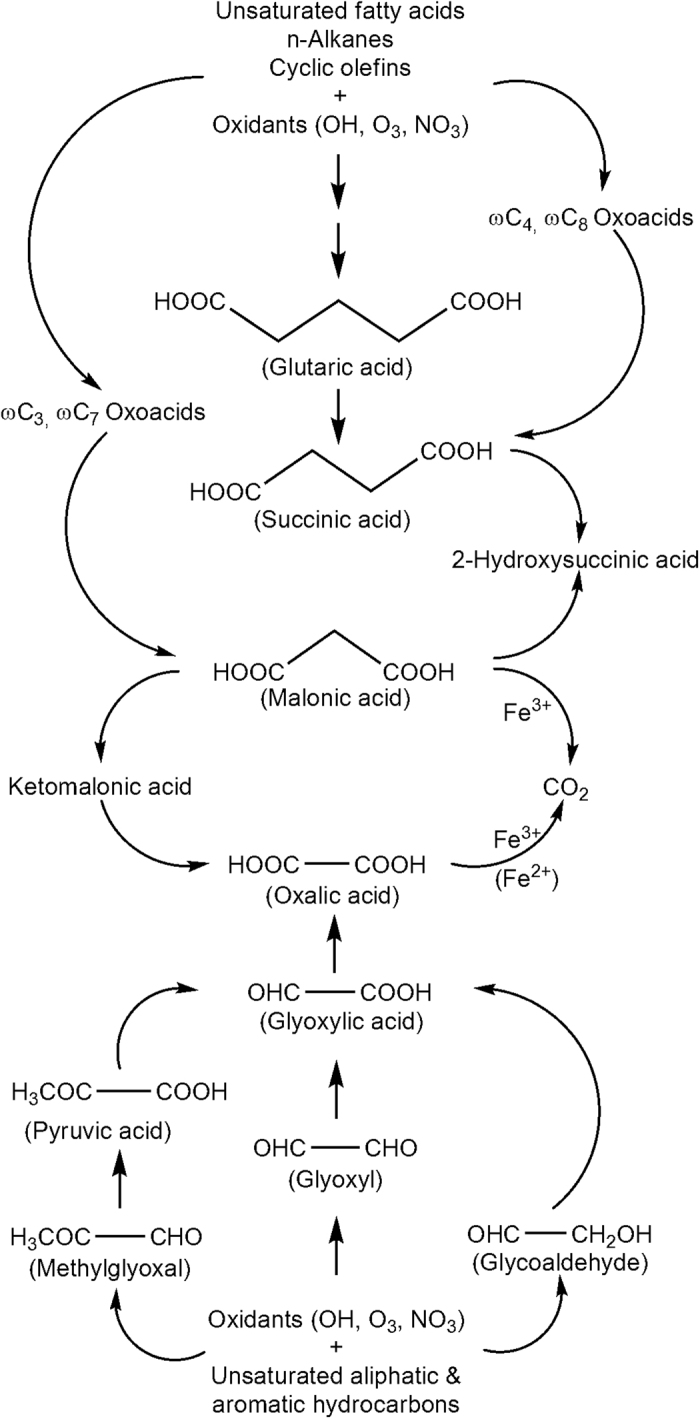
Possible photochemical oxidation pathways of diacids and related compounds in aqueous aerosols^14^,^16^,^20^,^21^,^27^,^28^,^29^,^40^,^41^,^42^.

In fact, Fe^2+^ and Fe^3+^ species are abundant in both AA (20.5 ng m^−3^ and 36.6 ng m^−3^, respectively) and BA (30.0 ng m^−3^ and 48.4 ng m^−3^, respectively)^19^, which could promote the photolysis of C_2_ and C_3_ diacids upon irradiation. The photolysis of C_2_ (and C_3_) in the presence of iron species could cause an isotopic fractionation and enrichment of ^13^C in the remaining C_2_ (and C_3_) diacids, because unidirectional reaction causes an enrichment of ^13^C in the remaining reactant^34^,^36^. Further the formation of Fe-oxalato (and malonato) complex and subsequent photolysis mainly depends on concentration ratios of Fe to oxalic (and malonic) acid rather than the UV light intensity^34^,^40^,^44^. Pavuluri and Kawamura^34^ reported an enrichment of ^13^C in authentic C_2_ diacid upon its photolysis in the presence of Fe^3+^ (and Fe^2+^) in aqueous phase in a laboratory experiment. Therefore, we interpret that an enrichment of ^13^C in C_2_ and C_3_ diacids with aging in AA and BA should have been mainly driven by their enhanced photochemical decomposition to CO_2_ (Fig. 2).

**Figure 3:**
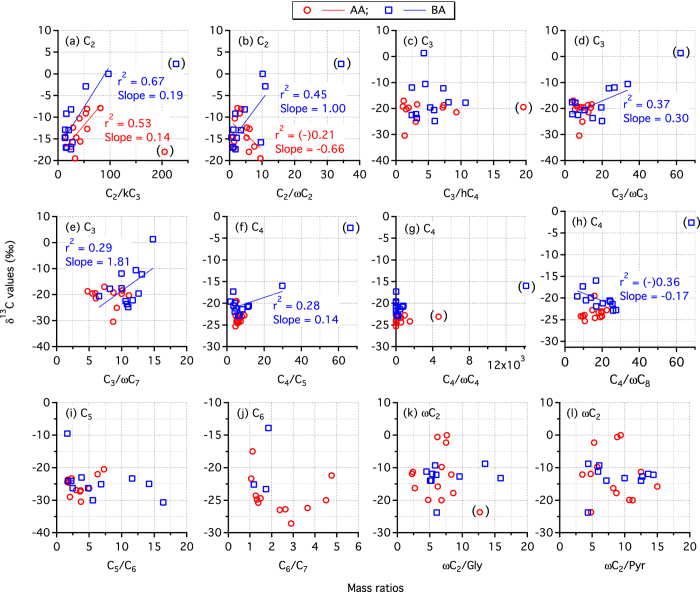
Scatter plots between mass ratios of selected diacids (C_2_-C_6_ and oxoacid (ωC_2_) to their immediate precursor compounds and δ^13^C of diacids (C_2_-C_6_) and ωC_2_. Please see text for abbreviations. Mass ratios data is obtained from Pavuluri *et al*.^19^. The outliers (1.75 × whisker length) that are not included in regression coefficient (r^2^) estimation are shown in brackets. Negligible r^2^ values (≤0.10) are not shown here.

It is well established that C_2_ and C_3_ diacids are produced by the photochemical breakdown of higher homologous diacids preferably via ketomalonic acid (kC_3_) and hydroxysuccinic acid (hC_4_), respectively. In addition, C_2_ is secondarily produced by the photochemical oxidation of unsaturated short-chain aliphatic, and aromatic hydrocarbons via ωC_2_ acid whereas C_3_ may be produced from unsaturated fatty acids and/or cyclic olefins via oxopropionoic (ωC_3_) or oxoheptanoic (ωC_7_) acids (Fig. 2)^19^,^21^,^24^. Hence, the trends of mass ratios of diacid species to their immediate precursor compounds could provide insights on the formation pathways and subsequent changes in δ^13^C of diacids. We found a linear relation between δ^13^C of C_2_ diacid and mass ratios of C_2_ to kC_3_ in both AA and BA (Fig. 3a) and also between δ^13^C of C_2_ and C_2_/ωC_2_ in BA but not in AA (Fig. 3b). On the other hand, although δ^13^C of C_3_ diacid showed no relation with mass ratios of C_3_ to hC_4_ in both AA and BA samples and with ωC_3_ and ωC_7_ acids in AA, it did show linear relations with C_3_/ωC_3_ and C_3_/ωC_7_ in BA (Fig. 3c–e). Such positive relations infer that enrichment of ^13^C in C_2_ and C_3_ is partly caused by the production from higher homologues and/or other precursors by sequential decarboxylation reactions (Fig. 2) because the substrate (i.e., reaction products retained in the same phase; particle or aqueous) are enriched with ^13^C, if some of the reaction products are volatile that are isotopically lighter^37^. This process is supported by the enhanced enrichment of ^13^C in C_2_ followed by C_3_ and C_4_ (Table 1).

On the contrary, a depletion of ^13^C in C_2_ diacid observed in few samples and a decrease in δ^13^C of C_3_ diacid during early stages of irradiation (Fig. 1a,b,j,k) can be attributed to an enhanced photochemical production of C_2_ and C_3_, whose δ^13^C are isotopically lighter than their precursor compounds such as oxoacids^32^,^33^,^36^ that are freshly produced from the primary precursors available in the original samples (Fig. 2). The reason for the observed inverse relation between δ^13^C of C_2_ and C_2_/ωC_2_ in AA is not clear, but we presume that photochemical degradation of C_2_ and its production from higher homologues could have played more important role in controlling δ^13^C than oxoacids in AA (Fig. 2). It is likely because the major precursors of ωC_2_; Gly and methylglyoxal (mGly), are mainly derived from biogenic emissions rather than anthropogenic sources^45^ and the formation pathway of ωC_2_ from mGly involves the decarboxylation reactions (Fig. 2), which causes an enrichment of ^13^C in the substrate (ωC_2_). Moreover, the ωC_2_ derived from aromatic hydrocarbons in AA might be enriched with ^12^C, rather than ^13^C, due to several unidirectional reactions.

Similarly, the production of C_3_ from C_4_ diacid via hC_3_ may be minor in both the samples as well as from oxoacids (ωC_3_ and ωC_7_) in AA. It is likely because the photochemical oxidation of longer-chain diacids results in either the immediate lower homologue or different short-chain oxoacid and diacid species^46^ whereas ωC_3_ and ωC_7_ are mainly derived from fatty acids that originate from biogenic emissions^21^. On the other hand, δ^13^C of C_3_ could have more influenced by its photochemical degradation and transformations rather than the production processes (Fig. 2), because the removal of CO_2_ in the former two cases leads to a significant enrichment of ^13^C in the remaining C_3_ diacid^37^. In the case of BA, production of C_3_ diacid via ωC_3_ and ωC_7_ acids (Fig. 2) should be significant because the abundances of fatty acids are higher (total concentration of C_8_-C_34_ fatty acids is 297 ng m^−3^) in BA than in AA (167 ng m^−3^)^19^.

The depletion and enrichment of ^13^C in C_4_-C_6_ diacids during early (up to 24~36 h) and later stages of irradiation, respectively, except for few cases, (Fig. 1c–e,l–n; Table 1) can also be attributable to the enhanced photochemical production from the first generation products, which are originally present in the samples and subsequent degradation to lower homologues (Fig. 2). However, plots between δ^13^C of C_4_ and mass ratios of C_4_ to C_5_ and oxooctanoic (ωC_8_) acid in AA, and oxobutanoic (ωC_4_) acid, and those between δ^13^C of C_5_ and C_5_/C_6_ and δ^13^C of C_6_ and C_6_/C_7_ in both the AA and BA samples are highly scattered (Fig. 3f–j), suggesting that production of C_4_-C_6_ diacids from their immediate higher homologues (and other precursors such as long-chain oxoacids), except for C_4_ in BA, may be insignificant in controlling δ^13^C of C_4_-C_6_.

Further, a significant enrichment of ^13^C in C_4_ and C_6_ in BA and AA, respectively, during the later stages of irradiation (Fig. 1) can be attributable to the photochemical breakdown to lower homologues (Fig. 2), because their concentrations were significantly low in those samples (Fig. 1l,e). The differences in δ^13^C trends of C_4_-C_6_ diacids between AA and BA (Fig. 1c–e,l–n) should have been driven by a significant difference in the abundances of their precursors in the original samples. For example, fatty acids are abundant in BA (297 ng m^−3^) than in AA (167 ng m^−3^)^19^. It is also important to note that depletion of ^13^C in C_4_-C_6_ in irradiated samples compared to that in non-irradiated samples (Fig. 1c–e,l–n; Table 1) should be a result of the enhanced production from the primary precursors that are originally present in the samples rather than the transformations of C_4_-C_6_ to their lower homologues (Fig. 2).

Although δ^13^C of C_9_ diacid were measured in few samples during early stages of irradiation, an enrichment of ^13^C was found in early stage of aging of AA (Fig. 1f), which can be attributed to photochemical degradation of C_9_ to lower homologues. In contrast, a depletion of ^13^C in C_9_ was found with the aging of BA as well as in 12 h irradiated sample of AA (Fig. 1f,o), which might be involved with simultaneous photochemical production of C_9_ diacid from its primary and first generation precursors such as fatty acids and ωC_9_ acid^21^. We found an enrichment of ^13^C in Ph with aging in both AA and BA (Fig. 1g,p), which can be explained by the enhanced photochemical degradation.

We also found an enrichment of ^13^C in ωC_2_ acid, except for few samples in BA, and Gly with aging (Fig. 1h,i,q,r). This increase may be caused by fragmentation reactions of their precursors (e.g., isoprene oxidation products, Fig. 2) that results in an enrichment of ^13^C in the remaining ωC_2_ and Gly in the same phase (i.e., particle/aqueous)^37^. Moreover, the breakdown of ωC_2_ and Gly to other oxygenated species (Fig. 2) on prolonged irradiation could also cause an enrichment of ^13^C in the remaining ωC_2_ and Gly^36^. It is noteworthy that the relations between δ^13^C of ωC_2_ and mass ratios of ωC_2_ to its precursors (Gly and pyruvic acid) are not linear but rather scattered (Fig. 3k,l). Simultaneous production of ωC_2_ from various precursors and further oxidation to C_2_ may cause a complicated behavior of the intermediate species (ωC_2_) in terms of stable isotopic composition (Fig. 2). As discussed in the cases of diacids, the differences in the abundances of precursor compounds in AA and BA might cause the variations in trends of δ^13^C of ωC_2_ and Gly between AA and BA samples. In fact, concentrations of ωC_2_ and Gly were significantly higher in BA than in AA during the early stages of irradiation (Fig. 1h,i,q,r), suggesting that their formation is more prominent than degradation in BA compared to that in AA because the original BA sample contains a large amount of organics (9820 ng m^−3^ of organic carbon) than AA (6400 ng m^−3^)^19^.

Thus the observed changes in δ^13^C of diacids, ωC_2_ and Gly. with aging in aqueous AA and BA were similar, except for few cases, which infer that the photochemical formation and degradation pathways of diacids and related compounds are almost same irrespective of their precursors origin and the δ^13^C of diacids and related compounds can be used as a proxy for aging. However, the differences in δ^13^C between AA and BA for some cases should have been driven by the differences in the abundances of diacids and related compounds as well as their precursor compounds in the non-irradiated AA and BA samples, which cause the differences in rates of their formation and/or degradation. In fact, diacids and related compounds are more abundant in non-irradiated AA than in BA whereas, organic carbon content that contains several precursors of those compounds is higher in BA than AA^19^. It is also of important to note that a significant formation of oligomers of carboxyl and carbonyl compounds in our experiment is unlikely because photolysis of oligomers should be more prominent during the prolonged aging of organics for several hours to days^11^,^13^.

Finally, it is noteworthy that a general trend of increased δ^13^C of C_2_ to C_4_ diacids with aging in both AA and BA samples are consistent with those reported in ambient aerosols from the western Pacific^38^, Sapporo, northern Japan^47^, and Chennai, India^39^, which are considered to be aged during long-range transport. Such consistency further supports that δ^13^C of diacids and related compounds can be used as a new proxy to trace aging of organic aerosols in the atmosphere. However, due to a lack of quantitative evaluation of oxidants (e.g., HO^•^) and complexity of photochemical processes of diacids and related compounds during the experiment, it is difficult to derive the KIE factors from this study, which is a subject of future research.

## Materials and Methods

**Figure 4:**
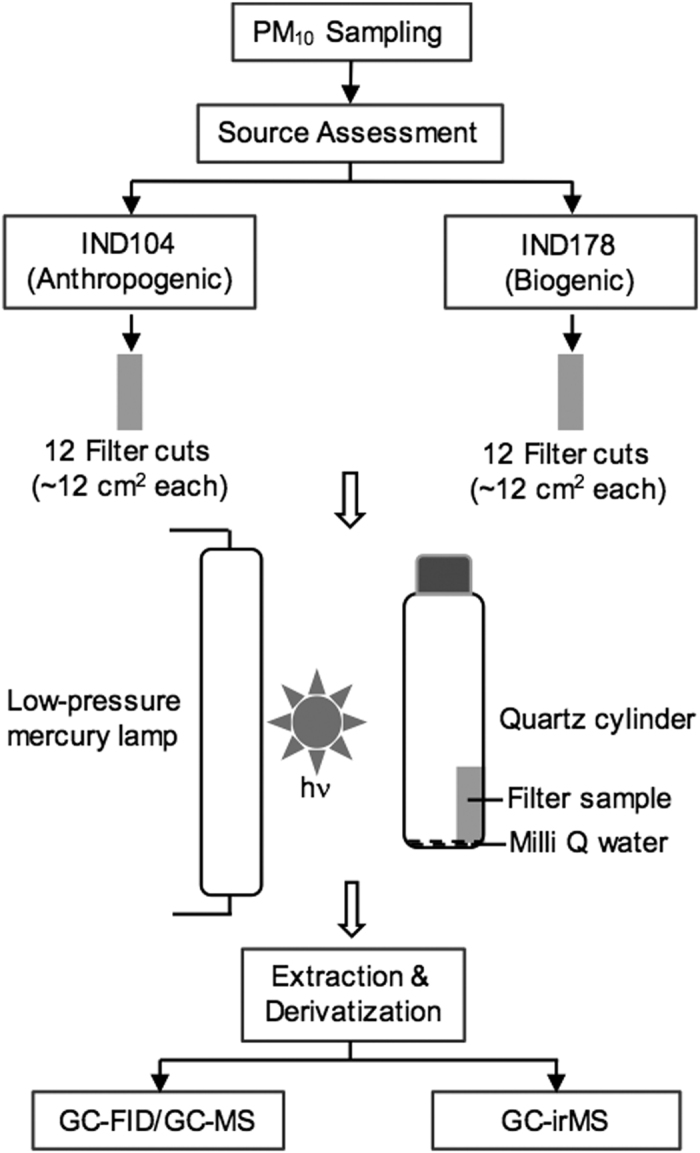
General scheme of the experimental procedure flow chart including the experimental setup for UV irradiation of atmospheric aerosol filter sample.

Batch UV irradiation experiments were conducted using two ambient aerosol (PM_10_) samples that were collected in winter on 28 January (IND104) and summer on 25 May (IND178) 2007 during daytime (06:00~18:00 h local time) from Chennai (13.03°N; 80.17°E), India. The procedure flow chart and schematic of the irradiation experimental setup are shown in Fig. 4. Details of sampling, chemical characteristics (source assessment) of aerosols and irradiation experiment are provided elsewhere^19^,^22^. Briefly, PM_10_ samples were collected using a high volume air sampler and pre-combusted (450 °C, 4 h) quartz fiber filters. The sample filter was placed in a pre-combusted glass jar with a Teflon-lined screw cap and stored in a dark freezer room at −20 °C prior to the experiment^22^.

As detailed by Pavuluri *et al*.^19^, backward air mass trajectories showed that air masses for IND104 originated from the north Indian subcontinent passing over the Bay of Bengal, where emissions from fossil fuel combustion and forest fires are significant. In contrast, the air masses for IND178 originated from the Arabian Sea passing over the south Indian subcontinent, where the emissions from combustion of biofuels and livestock are important. The concentrations of elemental carbon, organic carbon, hopanes (C_27_-C_35_, tracer for fossil fuel combustion), and fatty acids (C_8_-C_34_) and fatty alcohols (C_14_-C_34_) (biomarkers for biogenic emissions) were found to be 4810, 6400, 11.8, 167 and 93.3 ng m^−3^, respectively, in IND104 sample and 1810, 9820, 3.9, 297 and 178 ng m^−3^, respectively, in IND178 sample^19^. In addition, the trace metals that mainly originate from fossil fuel combustion (Cr, Pb and V) are higher (5.33, 133 and 9.60 ng m^−3^, respectively) by up to several times in IND104 than in IND178 (0.00, 39.9 and 0.00 ng m^−3^, respectively)^19^. Such chemical signatures indicate that IND104 sample is enriched with anthropogenic emissions whereas IND178 sample is with biogenic emissions. Hence, we considered that IND104 represents AA whereas IND178 represents BA.

Irradiation experiments of each sample were conducted for 0.5, 1.5, 3.0, 6.0, 12, 18, 24, 36, 48, 72, 96 and 120 h using a separate filter cut for every experiment. In each experiment, an aliquot (~12 cm^2^) of sample filter was placed vertically in a cleaned quartz reaction vessel (cylinder, 100 ml) with the sample surface facing to UV light and wetted with ~0.4 ml of ultra-pure organic free distilled water and then sealed with Teflon-lined screw cap under the ambient pressure. The filter samples were then irradiated with a low-pressure mercury lamp (Ushio, UL0-6DQ) that emits a UV light wavelength primarily at 254 nm and a minor peak at 185 nm in the presence of moisture in the reaction vessel.

The main objective of UV irradiation of aqueous aerosols (wetted sample filter) at 254 nm, rather than a solar spectrum, was to produce significant amount of hydroxyl radicals (HO^•^) via different pathways. Irradiation of aqueous aerosol at 254 nm induces the formation of O_3_ from the dissolved O_2_, followed by the generation of H_2_O_2_; the photolysis of H_2_O, NO_3_^−^, NO_2_^−^, H_2_O_2_, Fe(OH)^2+^ and certain organic compounds; and Fenton’s reaction of photochemically formed Fe^2+^ and H_2_O_2_^19^. Such HO^•^ sources are similar to those of atmospheric waters^48^ and the produced HO^•^ should be sufficient enough to act as the main oxidant in our experimental system because both the samples contain a large amount of Fe (2070 ng m^−3^ in AA and 553 ng m^−3^ in BA, including water-soluble iron)^19^ that could promote Fenton’s reaction upon UV irradiation. Moreover, O_3_, H_2_O_2_, HOO^•^ and NO_2_ formed in aqueous phase reactions may be partitioned into gas phase and generate the gaseous HO^•^ in the reaction cylinder that should be re-partitioned into aqueous phase^48^. However, we could not approximate the actual concentrations of HO^•^ in experiments, because we did not add any chemicals (e.g., a standard compound whose kinetics are known) into our experimental system in order to keep it as realistic as possible. We believe that the concentration of HO^•^ is almost constant in all experiments irrespective of the duration of each experiment, because the consumption and production of the HO^•^ could occur simultaneously during the experiment. Bateman *et al*.^11^ found no significant change in the concentration of H_2_O_2_, a major source of HO^•^, during aging of secondary OA of d-limonene in laboratory, which further supports our presumption.

Although we do not preclude a minor photolysis of some organic compounds present in aerosol samples by irradiation at 254 nm, it has been reported that targeted compounds of this study; low molecular weight diacids, oxoacids including pyruvic acid, and α-dicarbonyls such as mGly, have negligible absorbance at 254 nm and exhibit minimal photolysis, particularly when HO^•^ reactions of organics are significant^14^,^18^,^31^. The photolysis of organics by the absorbance of 185 nm light should also be insignificant during the experiment because the intensity of 185 nm peak is about 100 times lower than that of 254 nm and such small intensity of 185 nm light is mostly absorbed by water due to its high absorption coefficient (1.8 cm^−1^ at 25 °C)^49^. In addition, radiation at 254 nm has been reported to impose only a marginal photolysis of most inorganic species, except for nitrate, which is one of the HO^•^ sources^31^. The temperature of experimental system was maintained at 25 ± 1 °C to avoid any potential temperature effect on chemical reactions during the experiment.

Immediately after the irradiation on filter sample, diacids and related compounds in each sample were extracted and δ^13^C relative to Pee Dee Belemnite were determined using the method developed by Kawamura and Watanabe^50^. Briefly, irradiated filter sample was extracted with ultra-pure organic free distilled water (10 mL × 3) under ultra-sonication for 10 min. The extracts were concentrated to near dryness using a rotary evaporator under vacuum, and then diacids and related compounds were derivatized with 14% BF_3_/n-butanol at 100 °C to butyl esters and/or butoxy acetals. The derivatized samples were extracted with *n*-hexane and then determined for their peak identification and quantification using a capillary GC (HP 6890) and GC-MS (Thermo Trace MS)^20^,^22^,^51^, prior to the stable carbon isotope measurements.

After an appropriate amount of internal standard (*n*-C_13_ alkane) was spiked to a fraction of the derivatized sample, δ^13^C of the derivatives were determined using GC (HP6890) coupled to isotope ratio mass spectrometry (Finnigan MAT Delta Plus). δ^13^C of free diacids and related compounds in the sample were then calculated using an isotopic mass balance equation based on the measured δ^13^C of the derivatives and derivatizing agent (1-butanol)^49^. Each sample was analyzed in duplicate and an average is reported here. Difference in δ^13^C of free acids in duplicate analysis was generally below 1‰. However, the uncertainties for minor species were sometimes up to 1.5‰ and occasionally over 2‰. The experimental errors, including analytical errors, from replicate experiments (n = 3) conducted for 18 h irradiation of AA were found to be within 2‰, except for C_3_ diacid (3.2‰). No peaks were detected in the procedural blank of irradiation experiments (1.5 h and 6.0 h), except for a small peak for C_2_ and Ph acids, using a clean quartz filter.

## Additional Information

**How to cite this article**: Pavuluri, C. M. and Kawamura, K. Enrichment of ^13^C in diacids and related compounds during photochemical processing of aqueous aerosols: New proxy for organic aerosols aging. *Sci. Rep*. **6**, 36467; doi: 10.1038/srep36467 (2016).

**Publisher’s note:** Springer Nature remains neutral with regard to jurisdictional claims in published maps and institutional affiliations.

## Figures and Tables

**Table 1:** Stable carbon isotopic compositions (δ^13^C; ‰) of diacids and related compounds in non-irradiated IND104 (AA) and IND178 (BA) samples and average and median δ^13^C of those compounds in irradiated (0.5 to 120 h) AA and BA samples^a^.

Compound	AA			BA		
	Non-irradiated	Irradiated		Non-irradiated	Irradiated	
		Ave. ± SD	Median		Ave. ± SD	Median
Diacids						
Oxalic, C_2_	−19.5	−13.1 ± 3.59	−13.6	−15.8	−10.4 ± 6.85	−12.9
Malonic, C_3_	−21.4	−20.8 ± 3.99	−19.5	−20.6	−16.5 ± 7.70	−17.7
Succinic, C_4_	−22.8	−23.6 ± 1.46	−24.0	−19.6	−19.0 ± 5.54	−20.7
Glutaric, C_5_	−24.4	−25.4 ± 2.92	−25.5	−9.54	−25.8 ± 2.65	−25.0
Adipic, C_6_	−21.7	−24.6 ± 2.96	−25.0	−22.6	−18.6 ± 6.62	−18.6
Azelaic, C_9_	−30.6	−24.1 ± 3.38	−22.5	−30.2	−34.7 ± 2.42	−34.7
Phthalic, Ph	−34.6	−26.4 ± 3.99	−25.6	−28.7	−25.8 ± 2.29	−25.0
Oxoacid						
Glyoxylic, ωC_2_	−23.7	−11.5 ± 7.15	−12.0	−23.8	−11.9 ± 1.86	−12.2
α-Dicarbonyl						
Glyoxal, Gly	−9.50	−8.71 ± 19.5	−9.68	−20.8	−11.4 ± 11.7	−14.3

^a^δ^13^C data, except for C_5_ and Gly, in non-irradiated samples is obtained from Pavuluri *et al*.^39^; Ave.: Average; SD: Standrad Deviation.

**Table 2:** Correlation coefficients (r) between δ^13^C values and concentrations of diacids and related compounds in irradiated (together with non-irradiated) AA and BA samples.

	AA	BA
C_2_	0.36	0.58
C_3_	0.07	0.42
C_4_	0.01	0.25
C_5_	0.11	0.41
C_6_	0.68	0.81
C_9_	0.35	0.97
Ph	0.94	0.38
ωC_2_	0.19	0.17
Gly	0.19	0.06
